# Prognostic RNA-editing signature predicts immune functions and therapy responses in gliomas

**DOI:** 10.3389/fgene.2023.1120354

**Published:** 2023-02-08

**Authors:** Yi He, Xingshu Zhang, Sen Zhang, Yi Zhang, Bo Xie, Meng Huang, Junjie Zhang, Lili Shen, Wenyong Long, Qing Liu

**Affiliations:** ^1^ Department of Neurosurgery, Xiangya Hospital, Central South University, Changsha, Hunan, China; ^2^ National Clinical Research Center for Geriatric Disorders, Xiangya Hospital, Central South University, Changsha, China; ^3^ Department of Neurosurgery, People’s Hospital of Dengzhou, Dengzhou, Henan, China; ^4^ Guangdong Provincial People’s Hospital, Guangdong Academy of Medical Sciences, Guangzhou, China; ^5^ Guangdong Cardiovascular Institute, Guangzhou, China; ^6^ Department of Thoracic Surgery, Xiangya Hospital, Central South University, Changsha, Hunan, China

**Keywords:** RNA editing, glioma, prognosis, immune response, drug sensitivity

## Abstract

**Background:** RNA-editing refers to post-transcriptional transcript alterations that lead to the formation of protein isoforms and the progression of various tumors. However, little is known about its roles in gliomas.

**Aim:** The aim of this study is to identify prognosis-related RNA-editing sites (PREs) in glioma, and to explore their specific effects on glioma and potential mechanisms of action.

**Methods:** Glioma genomic and clinical data were obtained from TCGA database and SYNAPSE platform. The PREs was identified with regression analyses and the corresponding prognostic model was evaluated with survival analysis and receiver operating characteristic curve. Functional enrichment of differentially expressed genes between risk groups was performed to explore action mechanisms. The CIBERSORT, ssGSEA, gene set variation analysis, and ESTIMATE algorithms were employed to assess the association between PREs risk score and variations of tumor microenvironment, immune cell infiltration, immune checkpoints, and immune responses. The maftools and pRRophetic packages were used to evaluate tumor mutation burden and predict drug sensitivity.

**Results:** A total of thirty-five RNA-editing sites were identified as prognosis-related in glioma. Functional enrichment implied variation of immune-related pathways between groups. Notably, glioma samples with higher PREs risk score exhibited higher immune score, lower tumor purity, increased infiltration of macrophage and regulatory T cells, suppressed NK cell activation, elevated immune function score, upregulated immune checkpoint gene expression, and higher tumor mutation burden, all of which implied worse response to immune therapy. Finally, high-risk glioma samples are more sensitive to Z-LLNle-CHO and temozolomide, while the low-risk ones respond better to Lisitinib.

**Conclusion:** We identified a PREs signature of thirty-five RNA editing sites and calculated their corresponding risk coefficients. Higher total signature risk score indicates worse prognosis and worse immune response and lower sensitivity to immune therapy. The novel PREs signature could help risk stratification, immunotherapy response prediction, individualized treatment strategy-making for glioma patients, and development of novel therapeutic approaches.

## 1 Introduction

Gliomas are the most common primary brain tumors with marked heterogeneity, aggressiveness, and poor prognosis. Currently, even with standard treatments, namely a combination of surgery, radiotherapy, and chemotherapy, most glioblastoma (WHO grade Ⅳ glioma) will recur due to chemo- and radio-resistance and patients usually succumb quickly to the disease, with a five-year-survival rate around 5% (Ostrom et al., 2018a; Ostrom et al., 2018b; Wesseling and Capper, 2018). Advances in genomics, immunology, and many other disciplines have brought various experimental treatments, including targeted therapy and immunotherapy, that promise new avenues for glioma therapy. Unfortunately, so far these therapies have not been able to achieve satisfactory results (Qi et al., 2020; Yang et al., 2022). Therefore, it is of great significance to further explore the internal mechanism of glioma to discover new therapeutic targets and avenues.

Brain has long been considered an immune-privileged site for lacking traditional lymphatic systems. But recent findings showed that brain can coordinate a robust immune response involving both of innate and adaptive immune systems (Bailey et al., 2006; Louveau et al., 2015). The glioma-specific immunosuppressive microenvironment contributed greatly to the poor prognosis of patients with glioma (DeCordova et al., 2020). The studies of tumor microenvironment (TME) and its immune components have led to new potential therapeutic options for many extracranial solid tumors including melanoma (Motzer et al., 2015; Weber et al., 2015). Nevertheless, preclinical trials of immune checkpoint inhibitors and vaccine treatments for glioma failed to yield satisfactory results. This failure could partly come from the highly immunosuppressive microenvironment, systemic immunosuppression, local immune dysfunction, and high tumor heterogeneity of glioma cells (Qi et al., 2020; Medikonda et al., 2021), which makes the TME and immune alterations promising research area for developing novel and potent treatments for glioma.

RNA-editing refers to post-transcriptional transcript alterations that lead to the expression of protein isoforms. It can alter adenine to inosine (A>I) or cytosine to uracil (C>U) by adenosine and cytidine deaminases, respectively (Licht and Jantsch, 2016; Nishikura, 2016). Recent studies highlight that in cancer cells widespread RNA-editing partly make the transcriptomes more complex than genomes (Han et al., 2015; Paz-Yaacov et al., 2015). RNA-editing contributed to the carcinogenesis of several cancer types, and help cancer cells to adapt to distinct disease states and microenvironments (Han et al., 2015; Licht and Jantsch, 2016; Nishikura, 2016; Baysal et al., 2017). Given that dynamic change of RNA-editing levels during tumor progression, and that edited transcripts have a limited life span, the functional impact of RNA-editing on cancer cells might be different than those of permanent genomic alterations (Baysal et al., 2017). However, the exact roles of RNA editing in the progression of gliomas are not systematically studied yet, and its correlation with tumor microenvironments, immune function, and therapy response remain elusive.

In this study, as shown in the workflow in Supplementary Figure S1, we aim to identify the prognosis-related RNA-editing sites (PREs) in glioma to construct a prognostic signature model, and investigate the association between the risk signature and TME, tumor mutation burden (TMB) and chemotherapy sensitivity. This may shed light on the pathogenic mechanism, prognosis predication, risk stratification, and therapeutic strategy-making for patients with glioma.

## 2 Materials and methods

### 2.1 Data acquisition

The RNA sequencing, clinical information, and single nucleotide variation data of low-grade glioma (LGG) and glioblastoma (GBM) were obtained from TCGA database (https://portal.gdc.cancer.gov/, accessed 10 Aug 2022). RNA editing site data were acquired from SYNAPSE platform (https://www.synapse.org/#, accessed 10 Aug 2022).

### 2.2 Manhattan plot

Manhattan plot is a type of scatter plot usually used to display data with a large number non-zero amplitude data-points in genome-wide association studies (Reed et al., 2015). In this study we constructed Manhattan plot with “CMplot” package to display the overall RNA-editing landscape in gliomas. *p*-value is calculated with uni-variate cox regression analysis of the RNA-editing site profile and clinical survival data from TCGA. The cutoff value of top significant RNA editing sites is set at −log10p > 20.

### 2.3 Construction and validation of the RNA-editing prognostic signature

All patients were randomly divided into training or validation groups in a 6:4 ratio. In the training cohort, univariate cox analysis and the least absolute shrinkage and selection operator (LASSO) cox regression were applied to screen candidate PREs, which further underwent multivariate cox regression to evaluate their contribution to patient prognosis. RNA-editing sites with *p* < 0.0001 were considered as significantly prognosis-related. Based on multivariate regression coefficient and corresponding editing levels of those thirty-five PREs, prognostic risk score of each sample was calculated as the formula:

$$Riskscore=\overset{n}{\underset{i=1}{\sum }}X_{i}\times \beta_{i}$$

where n, Xi, and βi represent the total number, FPKM value and the corresponding regression coefficient of each RNA-editing sites. After calculation of total risk score for each sample, the median risk score of the training cohort was set as the cutoff value to divide the high-/low-risk groups for both of the training and the testing cohorts. Higher and lower risk scores imply overall alteration in PREs editing levels. The following prognosis and immune related analyses were performed on this basis.

Pheatmap package was utilized to show the expression pattern of PREs between 2 risk groups. Association between survival status and risk score was tested with scatter plots, and Kaplan-Meier analysis was utilized to examine the overall survival (OS) difference between risk groups (median risk score of the training cohort was set as the cutoff value to divide the high-/low-risk groups for both of the training and the testing cohorts). Through incorporation of risk score and other clinical factors, a nomogram was constructed to predict prognosis of a certain patient with given genomic and clinical characteristics. Thereafter, receiver operating characteristic (ROC) curve was applied to evaluate the prognosis prediction efficacy of the risk model and nomogram.

The R packages used in this section for data analysis include: “survival”, “ggplot2”, “caret”, “glmnet”, “dplyr”, “ggalluvial”, “survminer”, “pheatmap”, “timeROC”, “tidyverse”, “ggExtra”, “pec”, and “rms”.

### 2.4 Correlation between RNA-editing sites and corresponding RNA expression

The correlation relationship between RNA-editing and corresponding RNA expression levels was studied with Pearson’s correlational analyses incorporating the transcriptome data and the RNA-editing data. RNA-editing sites with significant correlation with RNA expression (correlation coefficient >0.3 or <−0.3) were further examined for their clinical relevance and prognosis impact.

### 2.5 Functional enrichment analysis

The significant differentially expressed genes (DEGs, |logFC| > 1 and *p* < 0.05) between high- and low-risk groups were identified with “limma” package and used for functional enrichment of gene ontology (GO) and Kyoto encyclopedia of genes and genomes (KEGG) pathways. To eliminate the subjective bias from artificial setting of significance threshold, we further performed gene set enrichment analysis (GSEA) with all expressed genes to explore the potential action mechanism of PREs in glioma.

R packages used for analysis and results visualization include “limma”, “org.Hs.eg.db”, “DOSE”, “clusterProfiler”, “enrichplot”, “scatterplot3d”, “ggplot2”, “circlize”, “ggpubr”, “colorspace”, “stringi”, and “RColorBrewer”.

### 2.6 Association between risk scores and TME

To examine the relationship between RNA-editing risk signature and TME, “ESTIMATE” and “CIBERSORT” packages were used to calculated the TME scores and tumor purity of each glioma sample (Chen et al., 2018). The relative abundances of 22 immune cell types were calculated to illustrate the association between RNA-editing risk score and immune cell infiltration. Single sample gene set enrichment analysis (ssGSEA) and gene set variation analysis (GSVA) was performed with “limma”, “GSEAbase”, and “GSVA” packages to investigate the enrichment variation of 29 immune-related functional gene sets between high- and low-risk groups. Besides, correlation between risk score and immune checkpoint genes (ICGs) were determined with “limma” package. Results visualization was carried out with “ggpubr”, “ggExtra”, “corrplot”, and “ggplot2” packages.

### 2.7 Analysis of association between risk score and TMB

TMB, namely the total number of somatic mutations of each sample, was calculated with “maftools” package, demonstrated with waterfall plot analysis, and combined with risk score for survival analysis using “survival” and “survminer” packages.

### 2.8 Prediction of therapy response

The relative efficacy of various drugs to each risk group was predicted with “pRRophetic” package and displayed in bubble plot, scatter plot and box plot with “ggplot2” package.

### 2.9 Statistical analysis

Wilcoxon test was employed for differential analysis and Spearman method was applied for correlation analysis between risk score and immune scores. All hypothesis tests were two-sided, with *p < 0.05, **p < 0.01 and ***p < 0.001. All other statistical analysis and data visualizations were carried out in R software (R version 4.1.2). Adobe Illustrator (CC 2017) was used for image processing.

## 3 Results

### 3.1 Identification of the PREs signature

For the construction and validation of the prognostic model, a total of 636 glioma samples with valid clinical and RNA-editing data were acquired and randomly divided into training or testing cohorts with a ratio of 6:4. The detailed characteristics of the two cohorts were summarized in Table 1, where there were no significant differences in the baseline characteristics between cohorts.

**TABLE 1:** Baseline characteristics of the glioma patient in training and testing cohorts.

Covariates	Type	Total	Test	Train	*p*-value
Age	≤ 65	552 (86.79%)	218 (85.83%)	334 (87.43%)	0.6405
Age	> 65	84 (13.21%)	36 (14.17%)	48 (12.57%)	
Gender	Female	273 (42.92%)	101 (39.76%)	172 (45.03%)	0.2182
Gender	Male	363 (57.08%)	153 (60.24%)	210 (54.97%)	
Grade	G2	234 (36.79%)	100 (39.37%)	134 (35.08%)	0.5421
Grade	G3	249 (39.15%)	96 (37.8%)	153 (40.05%)	
Grade	G4	153 (24.06%)	58 (22.83%)	95 (24.87%)	

**FIGURE 1:**
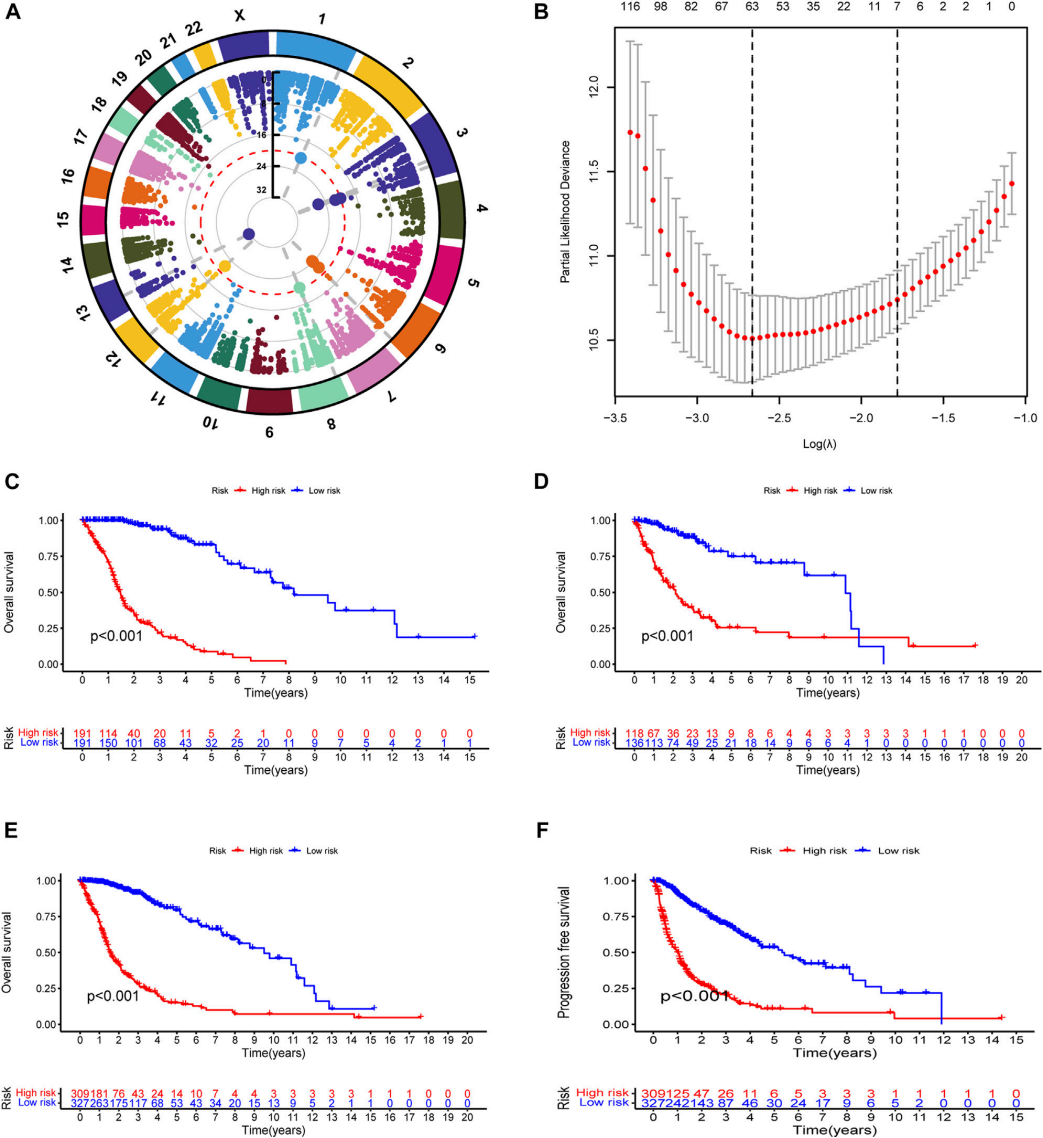
Construction and evaluation of prognostic RNA-editing signature in glioma: **(A)** Manhattan plot showing the global landscape of RNA-editing alterations with prognostic implications in gliomas. The number in the middle line indicates the −log10(p) for each RNA editing. The top significant RNA editings with −log10(p) > 20 were labeled in larger dot size. The grey dashed lines at from chromosomes to the middle demonstrated the chromosome location of those top significant RNA editings. **(B)** The cross-examination process to identify the optimal λ parameter. The x-axis showed the log(λ) value, and y-axis showed the corresponding likelihood deviation value. The vertical dashed line on the left corresponds to the optimal λ value; and the vertical dashed line on the right indicates the λ value of model with the evaluation index within 1 standard error of the optimal λ value, namely lambda.1se. The upper numbers are the number of edits enrolled for construction of model for each λ. In our model, the optimal log(λ) is around −2.6, with the corresponding likelihood deviation around −10.5 and 63 RNA editings were enrolled for prognostic examination. **(C–F)** Kaplan-Meier (K-M) survival analysis was performed to examine the prognostic value of the risk signature. Total risk score of each sample was calculated with editing levels of those thirty-five PREs and corresponding multivariate cox regression coefficients. Glioma samples were then divided into high- or low-risk groups based on risk score (median as the cutoff value). High-risk groups showed poorer survival in all of the training cohort **(C)**, testing cohort **(D)**, and overall glioma patients **(E)**, and shorter PFI in overall glioma patients **(F)**.

To explore the landscape of RNA-editing sites that are associated with prognosis in glioma, we applied cox regression analysis on the RNA-editing profile and prognostic survival data. Based on univariate cox regression results, Manhattan plot (Figure 1A) showed that gliomas have extensive genome-wide RNA-editing alterations with prognostic implications. Then, to correct overfitting of linear regression analysis, we perfored LASSO regression analysis (Figure 1B) and multivariate cox regression analysis. The optimal log(λ) is around −2.6, with the corresponding likelihood deviation around −10.5 and 63 RNA editing sites passed the threshold. After the regression analysis, 35 out of 63 RNA sites were identified as PREs in the training cohort with threshold of *p* < 0.0001 (Supplementary Table S1), and used for the construction of prognostic risk model.

### 3.2 Patients with lower risk score exhibited better prognosis

To test the reliability of the prognostic model, we used Kaplan-Meier (K-M) survival analysis to confirmed that in all of the cohorts (training, testing, and overall glioma patients), patients with lower risk score exhibited better prognosis (Figures 1C–E). A progression-free interval (PFI) advantage was also observed in the low-risk group of glioma patients (Figure 1F). To confirmed this correlation between risk score and patient prognosis, we further examined the survival status in the training, testing, and overall glioma patient populations. Compared with high-risk patients, a longer survival and a higher proportion of surviving patients were observed in the low-risk group (Supplementary Figure S2A–F). The expression patterns of PREs between risk groups were also displayed with heatmaps (Supplementary Figure S2G–I). Together, those results suggested that the PREs we identified are capable of stratifying glioma patients of different prognosis.

**FIGURE 2:**
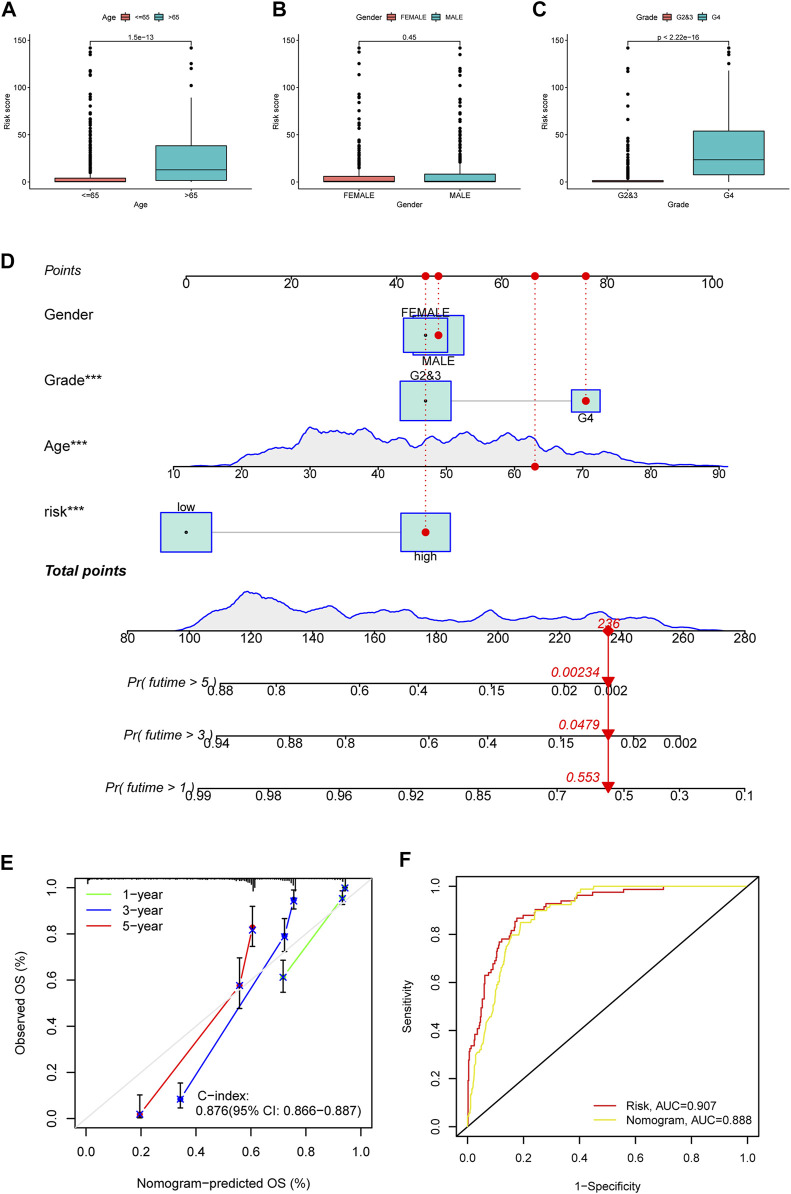
Clinical relevance of risk score, and construction and evaluation of nomogram. The risk score variation between patients of different ages **(A)**, genders **(B)** and tumor grades **(C)** were shown in box plots. **(D)** A nomogram to predict the 1-, 3-, and 5 year survival possibility of glioma patients. The blue lines of age and total points showed the distribution of the corresponding continuous variables in the data set used to establish the model. The points for each attribute are calculated according to the corresponding status and the matching location in the first row of points. The sum of points is then used to predict the 1-, 3-, and 5 year survival possibility for certain patients. An example is provided with red labels in the plot. **(E)** Calibration curve to evaluate the 1-, 3-, and 5 year OS prediction accuracy of nomogram. **(F)** ROC curve to examine the prognosis prediction efficacy of PREs risk model and nomogram for glioma patients.

Besides, we also explored the correlation between PRE risk scores and clinical features. Clinical relevance analysis revealed that older glioma patients with higher tumor grades have higher risk scores, but no difference was observed between genders (Figures 2A–C). Given that age and tumor grades both showed significant difference in total risk score, and older age generally comes with relatively higher tumor grades, there might be some confounding effects in these results, which would be well eliminated by subgroup analysis. Therefore, we further performed the subgroup analysis comparing the risk scores in old and grade 2,3 group vs. old and grade 4 group, as well as in young and grade 2,3 group vs. young and grade 4 group. Consistently, higher tumor grade samples have higher risk scores (Supplementary Figure S3). Thereafter, we integrated the clinicopathological variables and risk score to construct a nomogram to predict the 1-, 3-, and 5-year survival probabilities of glioma patients (Figure 2D). The calibration curve (Figure 2E) and ROC analysis (Figure 2F) both suggested this nomogram has an excellent performance in prognosis prediction.

**FIGURE 3:**
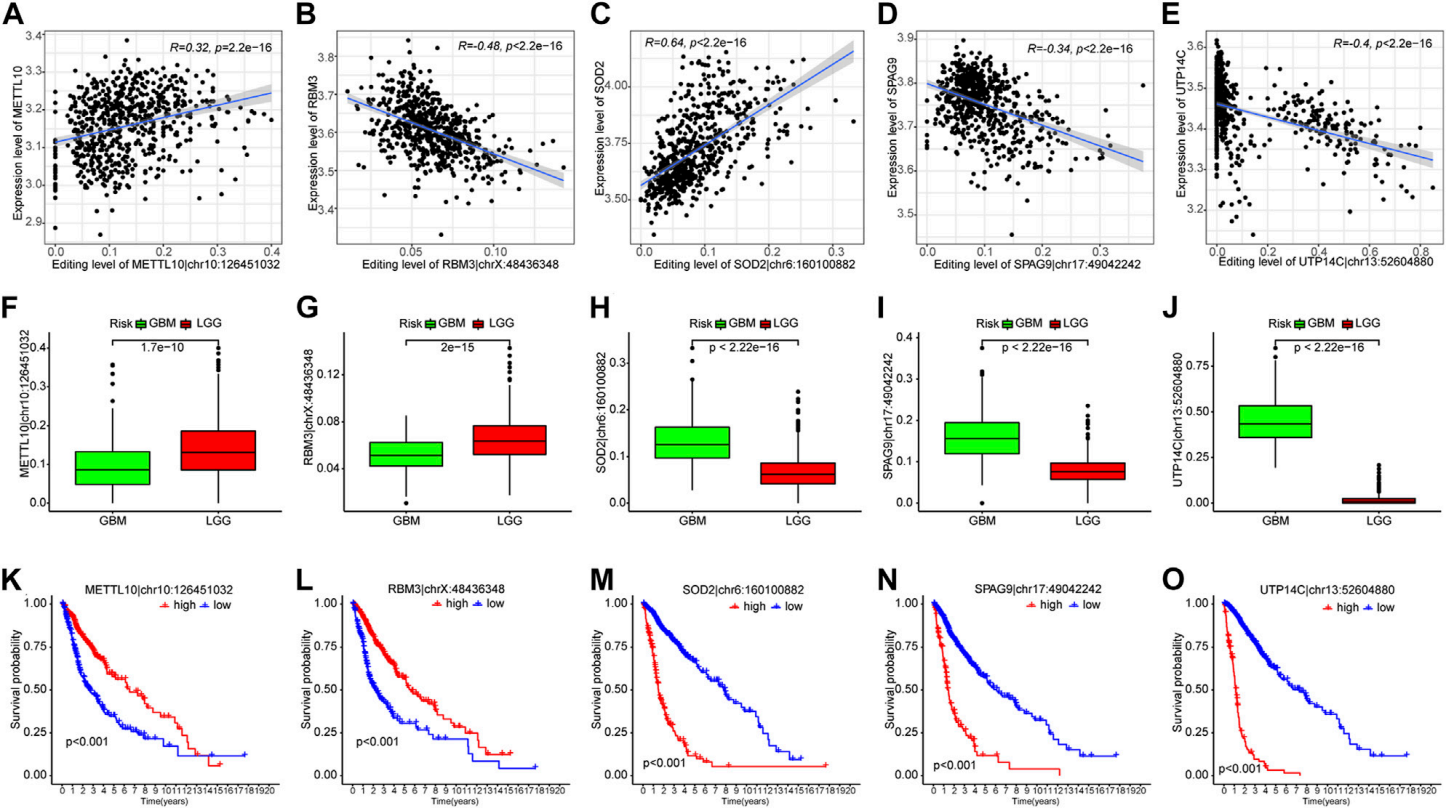
Five out of thirty-five PREs showed significant correlation with corresponding host genes. **(A–E)** Scatter plots showing the correlation between PRE editing levels and expression of corresponding host genes. **(F–J)** Difference of PRE editing levels between different grades of gliomas. **(K–O)** K-M survival analysis of those five RNA-editing in glioma patients (median of editing level as cutoff).

Given that analyzing all 35 PREs individually could be too complex and lengthy, to further narrow down the number of PREs for individual analysis, we performed a correlation analysis between the editing levels of PREs and the expression of the corresponding host genes. The results identified five PREs that significantly correlated with the expression levels of corresponding host genes (R <−0.3 or >0.3) (METTL10|chr10:126451032, RBM3|chrX:48436348, SOD2|chr6:160100882, SPAG9|chr17:49042242, and UTP14C|chr13:52604880) (Figures 3A–E). The following clinical relevance analysis and survival analysis showed that these five sites have significantly different editing levels between different grades of glioma (Figures 3F–J) and are closely related to the clinical prognosis of patients (Figures 3K–O).

The above results suggest that the PREs risk signature successfully predicted prognosis and could aid in the risk stratification of patients with glioma. RNA-editing sites, especially the PREs, may be involved in the oncological behavior of gliomas.

### 3.3 Functional enrichment analysis implied alterations of morphogenesis and immune pathways between different risk groups

**FIGURE 4:**
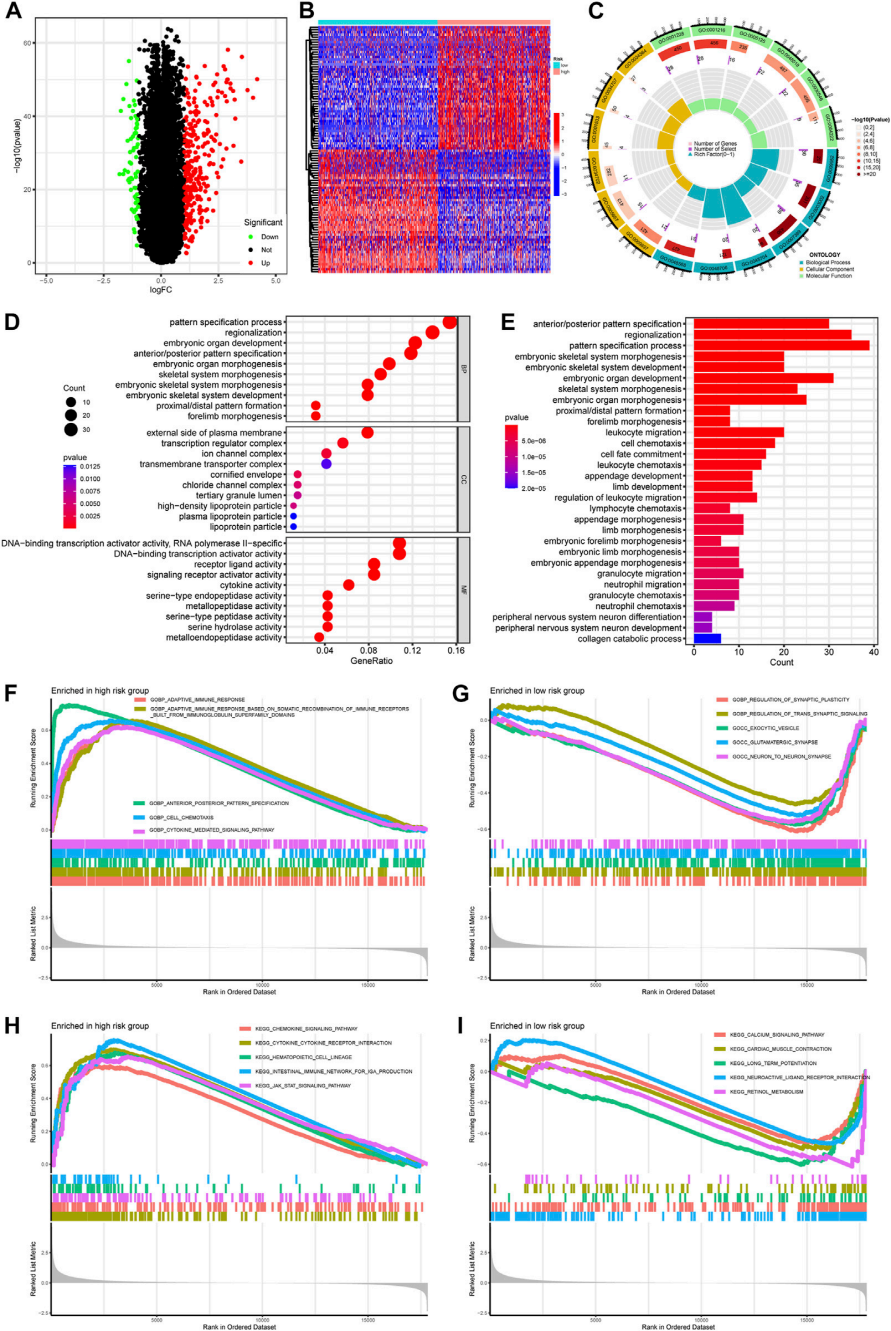
Pathway enrichment comparing gene expression profiles between high- and low-risk groups. **(A)** Volcano plot showing the DEGs between risk groups. Significance threshold was set as |logFC| > 1 and padj <0.05. Genes that are significantly upregulated or downregulated in high-risk group were labeled as red or green, respectively. **(B)** Heatmap showing the expression pattern of top 50 DEGs up- or downregulated in high-risk group. **(C,D)** GO enrichment of DEGs. **(E)** KEGG enrichment of DEGs. **(F,G)** GSEA enrichment of GO terms comparing high- and low-risk glioma samples. **(H,I)** GSEA enrichment of KEGG terms comparing high-and low-risk glioma samples. Abbreviation: GO, gene ontology; BP, biological process; MF, molecular function; CC, cellular component; KEGG, Kyoto Encyclopedia of Genes and Genomes; GSEA, gene set enrichment analysis.

To explore the potential biological processes and pathways associated with PREs, we first performed functional enrichment analyses on the DEGs using both KEGG and GO terms. With a significant threshold of adjusted. *p* < 0.05 and |log2(FC)|>1, 281 upregulated and 64 downregulated DEGs were identified (Figure 4A). Expression patterns of top 50 significant up- or downregulated DEGs were displayed in the heatmap (Figure 4B). GO enrichment analysis implied that DEGs were mainly involved in pathways of morphogenesis, transcription activity, and metallopeptidase activity in gliomas (Figures 4C, D). The KEGG results suggested that DEGs were closely associated with morphogenesis process and multiple immune-related pathways like leukocyte migration and chemotaxis (Figure 4E). Thereafter, to eliminate the subjective bias from artificial setting of significance threshold, we further performed gene set enrichment analysis (GSEA) with all expressed genes to explore the potential action mechanism of RNA-editing in glioma. Results suggested that many immune related pathways, such as adaptive immune response and chemokine signaling pathways, are significantly enriched in the high-risk group samples.

These results implied that PREs might affect the biological behavior of gliomas by altering the morphogenesis, immune response, and transcription activity. The glioma-specific immunosuppressive microenvironment is widely recognized for its contribution to the poor prognosis of glioma (DeCordova et al., 2020). Previous study proved that inhibition of adenosine-to-inosine editing could promote expression of immune response protein MAVS in GBM (Raghava Kurup et al., 2022), and RNA editing activity was reported to inhibit cell migration and proliferation of astrocytomas (Cenci et al., 2008). If the PREs identified in this study do be capable of altering the immune response of glioma, they might shed light on development of new therapeutic avenues for glioma patients.

### 3.4 PREs signature correlates with immune activation status in gliomas

**FIGURE 5:**
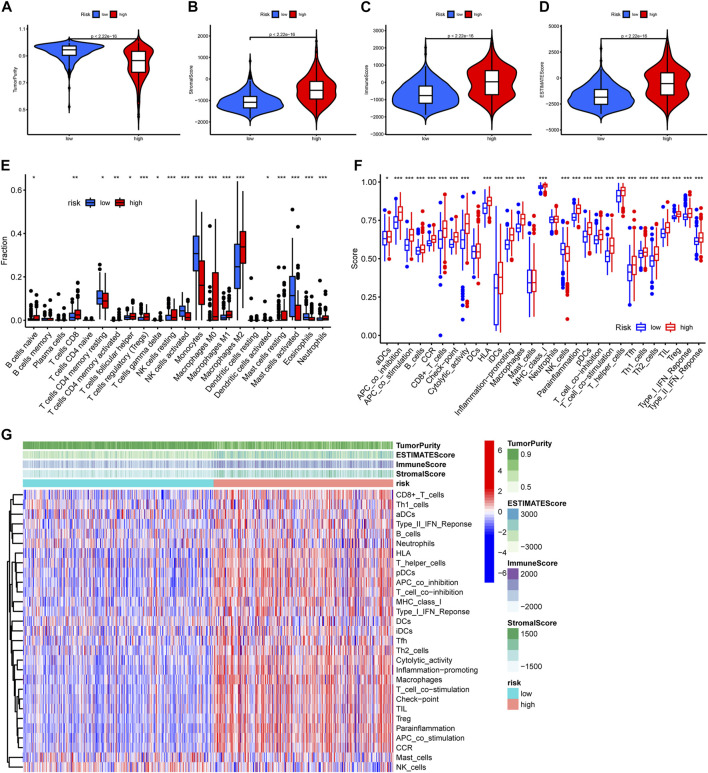
Glioma samples with different risk score possess different immune functions. **(A–D)** Comparison of tumor purity, stromal scores, immune scores, and ESTIMATE scores between the high- and low-risk groups. **(E)** The abundance variation of 22 immune cells in the high- and low-risk groups. **(F)** Enrichment differences of immune-function-related gene sets between high- and low-risk group glioma samples. **(G)** Heatmap exhibiting the immune function enrichment alteration between different risk group glioma samples. *: *p* < 0.05, **: *p* < 0.01, ***: *p* < 0.001.

TME has considerable impact on the treatment sensitivity and prognosis of glioma, therefore we examined TME variation between different risk groups to explore if PREs are associated with TME composition of glioma. As shown in Figures 5A–D, high-risk group glioma samples exhibited higher immune scores, stromal scores, ESTIMATE scores, and lower tumor purity than did the low-risk ones. Immune infiltration analysis revealed that the high-risk group glioma samples possess more abundant regulatory T cells (Tregs), macrophages, and resting NK cells, and have fewer monocytes and activating NK cells (Figure 5E). Additionally, immune function analysis suggested that compared to the low-risk glioma samples, the high-risk group ones exhibit higher scores in almost all immune function gene sets, and lower score in NK cell functions (Figures 5F, G). Tregs and macrophages are reported to contribute to the immune suppression environment of glioma, and the NK cells is known for its cell toxic and anti-tumor effects (Wei et al., 2020) (Fecci et al., 2006) (Zhang et al., 2016; Burger et al., 2019). Our findings implied that in glioma, the PREs may be associated with immune activation status of Tregs, macrophages and NK cells, which might be one mechanism of PREs’ prognostic impact in glioma.

### 3.5 PREs risk scores positively correlate with ICGs expression in glioma

**FIGURE 6:**
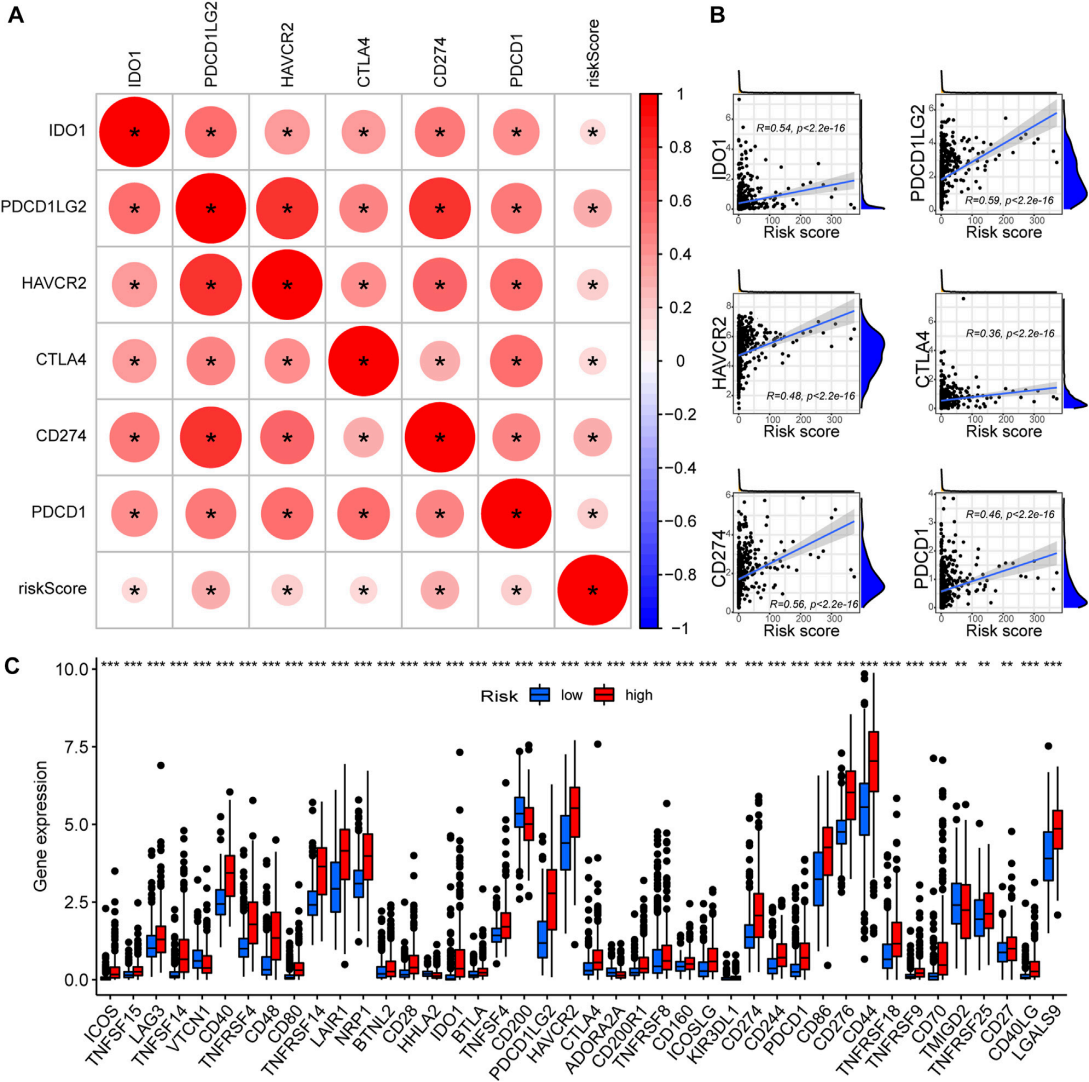
High-risk glioma samples possess elevated ICGs expression. **(A)** The correlation between critical ICGs and risk score; Red color indicates a positive correlation, and a darker color intensity and larger circle represent a stronger correlation. *: *p* < 0.05. **(B)** Scatter plots demonstrating the correlation between the risk scores and the expression levels of each critical ICGs. **(C)** The expression differences of 47 ICGs between the high- and low-risk group glioma samples.

As PREs risk scores are significantly associated with immune activation status in gliomas, we investigated if the risk scores are associated with the expression levels of ICGs. All six of the most critical ICGs that were examined, including CD274, CTLA4, HAVCR2, IDO1, PDCD1, and PDCD1LG2, exhibited a significant positive correlation with the risk scores in glioma samples (Figure 6A). The individual correlation scatter plots are exhibited in Figure 6B. Furthermore, we examined the expression differences of forty-seven main ICGs between risk groups, and the results revealed that most of the ICGs were highly expressed in the high-risk group glioma samples (Figure 6C). ICGs are commonly involved in immune tolerance and proliferation of tumors including glioma, and their blockage might inhibit the tumor progression (Ghouzlani et al., 2021). Our results suggest that the PREs risk scores positively correlate with ICGs expression. Some members of the PREs may contribute to the immune tolerance of glioma through altering the expression of ICG, and targeting them could be promising novel therapeutic approach.

### 3.6 Glioma samples with higher risk score exhibit higher overall TMB

**FIGURE 7:**
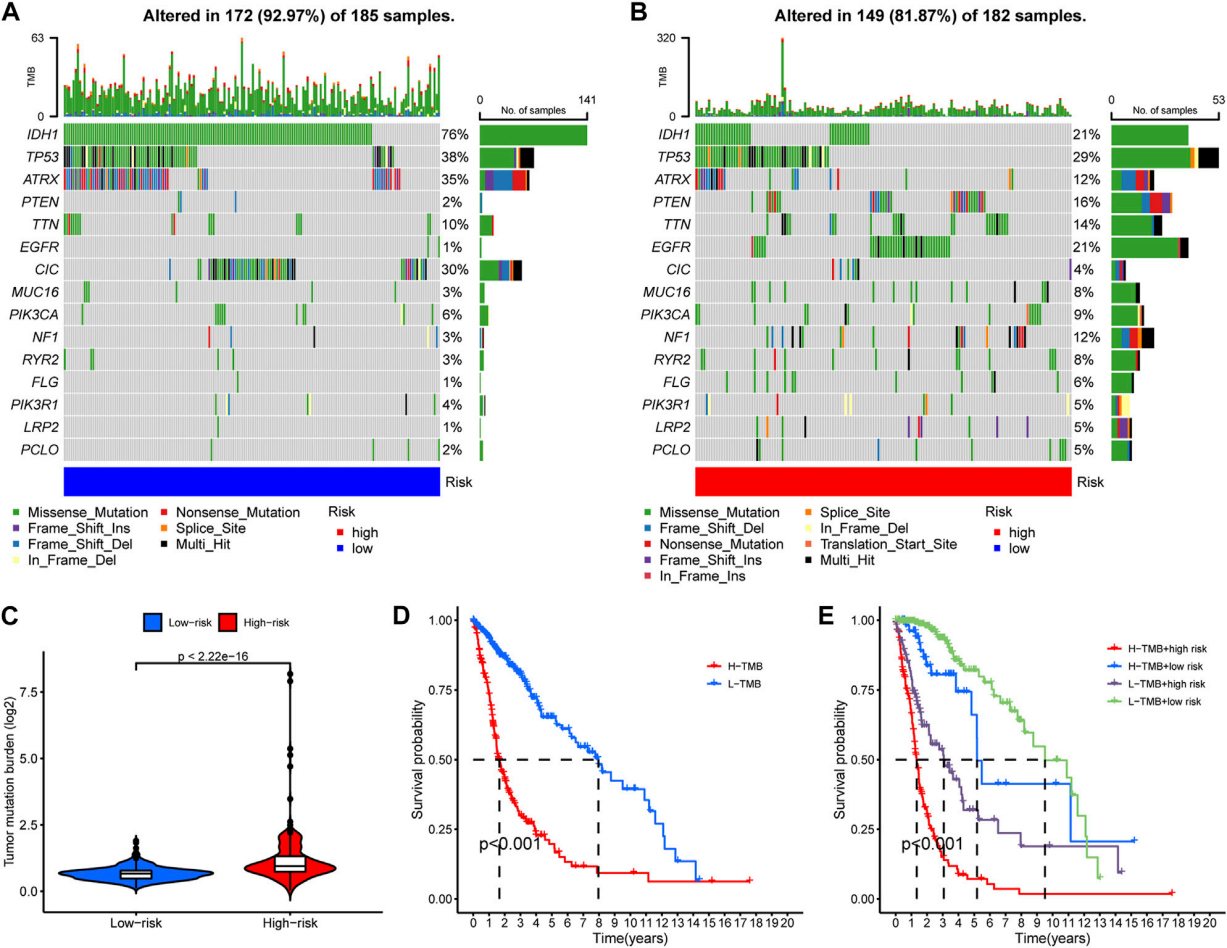
A general heavier tumor mutation burden (TMB) was observed in high-risk glioma samples and correlated with worse prognosis. **(A–B)** The occurrence frequency of the top 15 mutations in the high- and low-risk group glioma samples, respectively. **(C)** Overall TMB variation between the high- and low-risk groups. **(D)** K-M analysis to examine the OS difference between glioma patients with different levels of TMB. **(E)** Comprehensive survival analysis of glioma pa-tients incorporating both of the PREs risk score data and the TMB information.

We then compared the mutation landscapes variation between the high- and low-risk glioma samples. The low-risk group possessed higher rates of IDH1, ATRX and CIC mutations than did the high-risk group (76% vs 21%, 35% vs 12%, and 30% vs. 4%, respectively), but most of the other common mutations occurred more frequently in the high-risk group, especially for EGFR mutation (1% vs. 21%) (Figures 7A, B). We also compared the risk score of the samples with/without mutations of IDH1, ATRX, and CIC, and found that samples with those mutations have significantly lower risk score (Supplementary Figure S4). This might account for the elevated overall TMB in the high-risk group samples (Figure 7C). Then, to investigate the impact of TMB on the prognosis of glioma patients, we performed K-M analysis to examine the OS difference between glioma patients with different levels of TMB. Results indicated that glioma patients with higher TMB showed significantly shorter OS (Figure 7D). In addition, we integrated the RNA-editing risk scores with TMB data to analyze the prognostic impact of those two factors. The results suggested that patients with both lower risk and lower TMB exhibited the best prognosis compared to that of the other subgroups (Figure 7E). This inspired us to use overall TMB for prognosis prediction of patients with glioma, and the combination of the PREs risk score and TMB might possess a higher prognostic value.

### 3.7 Prediction of chemotherapy response in different risk groups

**FIGURE 8:**
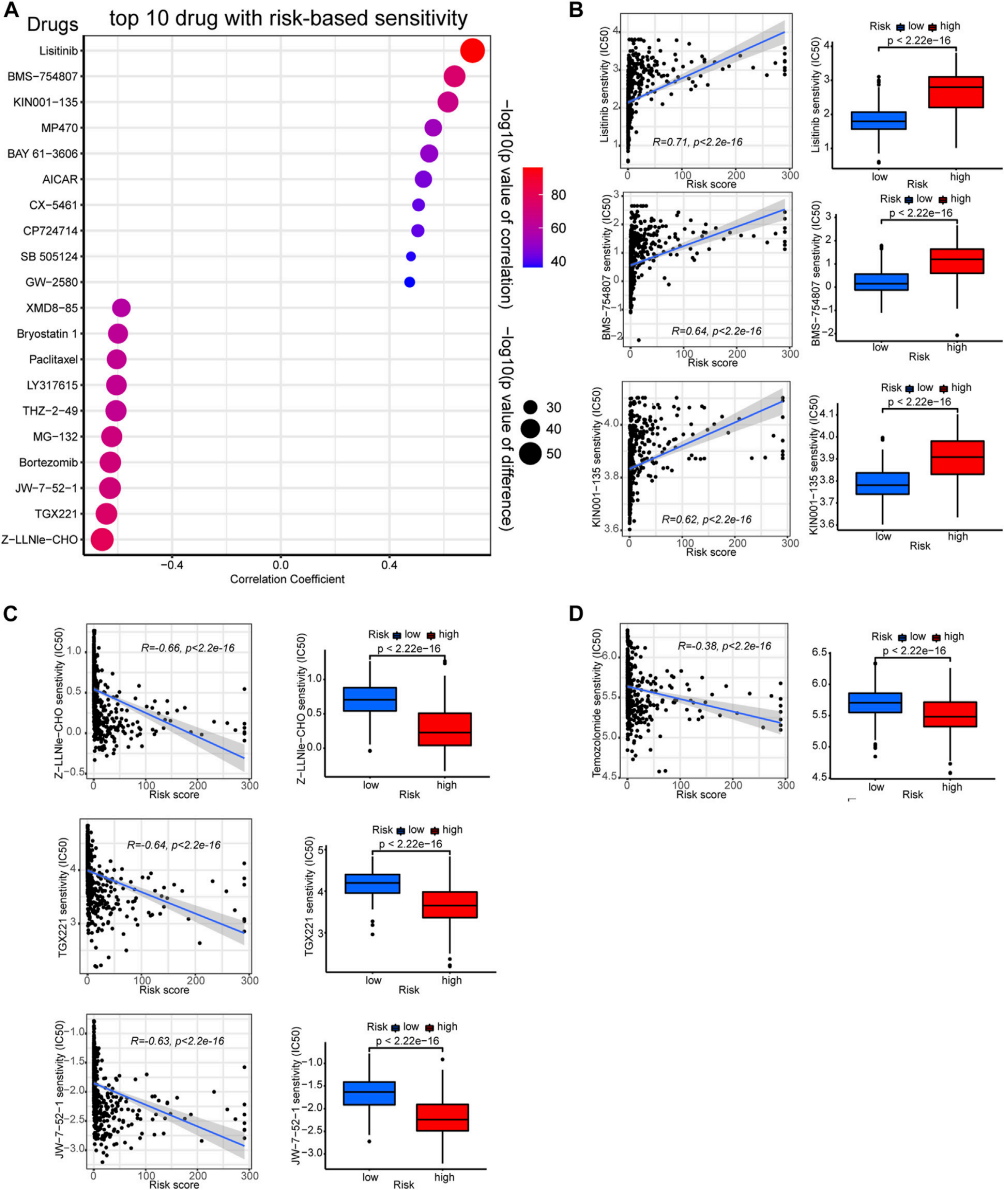
Correlation between the PREs risk score and chemotherapy sensitivity of gliomas. **(A)** Bubble plot displaying the top ten drugs with sensitivity preference to high- or low-risk group glioma samples, respectively. Correlation scatter plots and sensitivity difference box plots of drugs that are more effective to low-risk **(B)** or high-risk **(C)** glioma samples. Correlation scatter plots and sensitivity difference box plots of temozolomide **(D)**.

As previous studies indicated that TMB levels are associated with therapeutic response and sensitivity (Büttner et al., 2019), we further employed the “pRRophetic” algorithm to compare between risk groups the sensitivity differences of common chemotherapies. Certain drugs are more effective to low-risk group samples, while others exhibit higher efficacy to high-risk ones. The top ten sensitive drugs for the low- or high-risk groups are exhibited in the bubble plot (Figure 8A). The sensitivity correlation and IC50 differences of the top three sensitive drugs for each risk groups are also presented with scatter plots and box plots (Figures 8B, C). Low-risk group samples could be more sensitive to linsitinib, BMS−754807, and KIN001−135, while high-risk samples may be relatively more sensitive to drugs of Z-LLNle-CHO, TGX221, and JW-7-52-1. The preferential sensitivity of those drugs implied that different editing levels of PREs might come with different response to those drugs in glioma, and manipulating PREs might have synergetic effect with those drugs in glioma.

Besides, given that temozolomide is the most commonly used anti-glioma drug, we further examined its efficacy in each risk group. As presented in Figure 8D, high-risk glioma samples showed lower IC50 values for temozolomide, suggesting they could be more sensitive to it. Together, these results might shed light on the potential therapeutic strategy-making for patients with glioma.

## 4 Discussion

Gliomas are a group of lethal brain tumors with extremely poor prognosis. Even with complete standard therapy, patients generally die quickly of the disease due to therapy resistance and tumor recurrence (Ghotme et al., 2017). The glioma-specific immunosuppressive microenvironment contributed greatly this doomed prognosis (DeCordova et al., 2020), but preclinical trials of treatments against it have failed to yield satisfactory results. RNA-editing contributed to the carcinogenesis of several cancer types (Han et al., 2015; Baysal et al., 2017). However, in gliomas the exact correlation between RNA-editing and carcinogenesis, tumor microenvironments, immune function, and therapy response remain elusive.

In this study, utilizing public RNA-editing and clinical data of glioma patients, we identified thirty-five PREs in glioma (Supplementary Table S1) and constructed a risk stratification model accordingly. Survival, clinical relevance and ROC analysis confirmed that the risk model can accurately predict the prognosis of patients with glioma. Consistently, RNA-editing has been reported to impact the prognosis of multiple solid tumors, including but not limited to uterine corpus endometrial carcinoma (Wu et al., 2021), hepatocellular carcinoma (Chen et al., 2020), and esophageal squamous cell carcinoma (Qin et al., 2014). But given the heterogeneity between tumors and organ-selective expression of genes, the specific RNA-editing that affect prognosis vary by tumor. The PREs signature presented in this study can not only provide novel prognostic prediction models for patients with glioma, but also offer potential targets to inhibit the progression of gliomas.

In regard to the underlying action mechanism of PREs in glioma, DEGs functional enrichment implied that the variation of PREs correlate with alterations in pathways of morphogenesis, transcription activity, and immune process. Immune response escape, tumor-promoting inflammation, and genome instability are known hallmarks of cancer (Hanahan and Weinberg, 2011). Accumulating evidence suggests that RNA-editing is involved in the alteration of the immune response and transcription activity in various tumors. RNA-editing impacts the mRNA abundance of immune response pathways in multiple cancers including breast invasive carcinoma, lung adenocarcinoma, prostate adenocarcinoma, (Chan et al., 2020). Inhibition of adenosine-to-inosine RNA-editing could promote expression of immune response protein MAVS (Raghava Kurup et al., 2022). RNA-editing enzyme ADAR1 could regulate R-loop formation and genome stability at telomeres in cervical cancer (Shiromoto et al., 2021). Similar to these studies, our results implied that PREs might impact the prognosis of glioma through altering pathways of immune response and transcription activity.

Functional enrichment analysis revealed that immune response alteration could be one of the potential action mechanisms of PREs in gliomas. However, the exact association between PREs levels and glioma immune response remains elusive. Our immunology-related analysis showed that high-risk glioma samples possess elevated TME immune scores, immune function scores, and ICG expression. Infiltrations of Treg and macrophage are promoted in high-risk group, while NK cell function is suppressed. Glioma-associated macrophages (GAMs) are the major immune cell population in gliomas with significant tumor-promoting effects (Wei et al., 2020). Tregs exert considerable immune-suppressive effects in gliomas, and generally increase with the reduction of anti-tumor CD4^+^ T cells fraction (Fecci et al., 2006). NK cells are well-established anti-tumor immune cells, whose activity is commonly suppressed by glioma TME. Activity restorations of NK cells through chimeric antigen receptor (CAR)-engineering are extensively studied for application in glioma immunotherapy (Zhang et al., 2016; Burger et al., 2019). The increased infiltration of macrophage and Treg, as well as the suppression of NK cell activation may partially account for the worse prognosis of the high-risk group patients. Besides, our results revealed that most ICGs were highly expressed in the high-risk group glioma samples. ICGs, particularly the key members such as CTLA-4 and PD-1, are profoundly involved in immune tolerance and proliferation of glioma cells, and their blockage might inhibit the progression of gliomas (Ghouzlani et al., 2021). Therefore, global upregulation of ICGs may also be partially responsible for the poor prognosis of patients in the high-risk group. Interestingly, in addition to tumor-promoting GAM and Treg cells, a majority of other immune functions, some of which might exert anti-tumor effect in other solid tumors, were also activated in the high-risk group. On the one hand, this coincided with the fact that RNA-editing could extend the classes of HLA presented self-antigens, which can be recognized by the immune system and boost immune function (Zhang et al., 2018). But meanwhile, this is contradictory to the fact that high risk group glioma patients have worse prognosis. Possible explanations include their functions may be overshadowed by GAM and Treg activation and NK cell suppression due to their lower abundance in gliomas, or these common tumor-suppressing immune components may be reprogrammed in gliomas to play different biological roles. However, the exact immune process activation status and their actual effects in glioma require further experimental verification.

Tumor heterogeneity, including genomic heterogeneity, contributes greatly to the therapy-resistance and poor prognosis of gliomas (Nicholson and Fine, 2021). As an important types of genomic heterogeneity, TMB has an essential impact on glioma prognosis. A pan-cancer study published in “Annals of oncology” reported that for cancers like glioma, where there is no correlation between CD8^+^ T cell levels and neoantigen loads, higher TMB is associated with tolerance to immune response and immunotherapy (McGrail et al., 2021). This is consistent with our finding that high-risk glioma patients possess higher overall TMB and worse prognosis. Additionally, the low-risk group glioma samples possessed more frequent mutations in IDH, ATRX and CIC genes, whose mutations possess anti-tumor effects in gliomas (Yan et al., 2009; Bettegowda et al., 2011; Qin et al., 2022). This may also partially explain the prognostic advantage in the low-risk group glioma patients. Together, PREs may be associated with the genome mutation landscapes and overall TMB levels in gliomas.

Previous study has shown that RNA-editing signature could be used to predict therapy response in tumors like advanced gastric cancer (An et al., 2021). Likewise, we used our PREs risk model to study the risk group-preferential sensitivity of various drugs against glioma samples, trying to identify some novel therapeutic options for glioma. Based on our results, low-risk group gliomas could be more sensitive to linsitinib, BMS−754807, and KIN001−135, while Z−LLNle−CHO, TGX221, and JW-7-52-1 may be more effective to high-risk gliomas. Additionally, temozolomide also exhibited higher sensitivity against high-risk glioma samples. Linsitinib is an insulin-like growth factor 1 receptor (IGF-1R) inhibitor that can inhibit the growth of diffuse midline glioma with H3K27M mutations (de Billy et al., 2022). BMS-754807 is also an IGF-1R inhibitor reported to prevent radiotherapy resistance in pediatric/youth high-grade gliomas (Simpson et al., 2020). KIN001−135 is a small-molecule inhibitor for multiple targets including TANK binding kinase 1, and is under preclinical trials for glioma treatment (Xia et al., 2018). Z-LLNle-CHO is a gamma-secretase inhibitor that can trigger cell death in leukemia and breast cancer (Han et al., 2009; Meng et al., 2011), but its biological roles have not yet been explored in glioma. TGX-221 is a selective inhibitor of p110β-PI3K that can block the activation of PKB/Akt pathway in PTEN-deficient cells. TGX-221 is reported to induce apoptosis and inhibit migration and invasion in glioblastoma cells (Yang et al., 2017). JW-7–52-1 is a PI3K/MTOR signaling pathway inhibitor that hasn’t been tested for effects in gliomas. Taken together, our findings identified some potential drugs that may be used in glioma therapy, and shed light on the possibility of using these drugs to target RNA-editing to inhibit glioma growth.

The current study still has some limitations. First, the research was based on bioinformatics analysis, so prospective analysis of real-world data is required to verify the robustness and clinical utility of the risk model. Additionally, for the underlying action mechanism of PREs in gliomas, we only explored correlations but did not experimentally verify causality. Further rigorous experimental validations are necessary for the elucidation of internal mechanisms.

## 5 Conclusion

In summary, we identified PREs in gliomas that could help to predict patient outcomes. PREs risk scores correlate with alterations of immune response, immune checkpoint expression, and TMB in gliomas. The identified PREs signature may contribute to the clinical risk stratification of glioma patients, but the specific mechanism of PREs in the context of glioma awaits further experimental study.

## Data Availability

The original contributions presented in the study are included in the article/Supplementary Material, further inquiries can be directed to the corresponding authors.

## Supplementary material

SUPPLEMENTARY TABLE S1 Thirty-five PREs and corresponding coefficients.

SUPPLEMENTARY FIGURE S1 The study workflow.

SUPPLEMENTARY FIGURE S2 The correlation between the PREs risk signature and the prognosis of the training cohort **(A, D, G)** , testing cohort **(B, E, H)**, or overall glioma patients **(C, F, I)**. **(A–C)** The risk score distribution of patients with gliomas. **(D–F)** Patients survival matched to risk score distribution results. **(G–I)** Heatmaps showing the expression pattern of PREs between risk groups.

SUPPLEMENTARY FIGURE S3 Subgroup analysis of risk score clinical relevance. The risk score variation between patients in old and grade 2,3 group vs old and grade 4 group **(A)** , as well as in young and grade 2,3 group vs young and grade 4 group **(B)** were shown in box plots.

SUPPLEMENTARY FIGURE S4 Risk score variation of the wild type or mutant samples about IDH1 **(A)**, CIC **(B)**, and ATRX **(C)** genes.
