# Supplementary figures and images for: Prognostic RNA-editing signature predicts immune functions and therapy responses in gliomas
Source: Front Genet. 2023 Feb 8;14:1120354. doi: 10.3389/fgene.2023.1120354 (PMC9945230; doi:10.3389/fgene.2023.1120354)

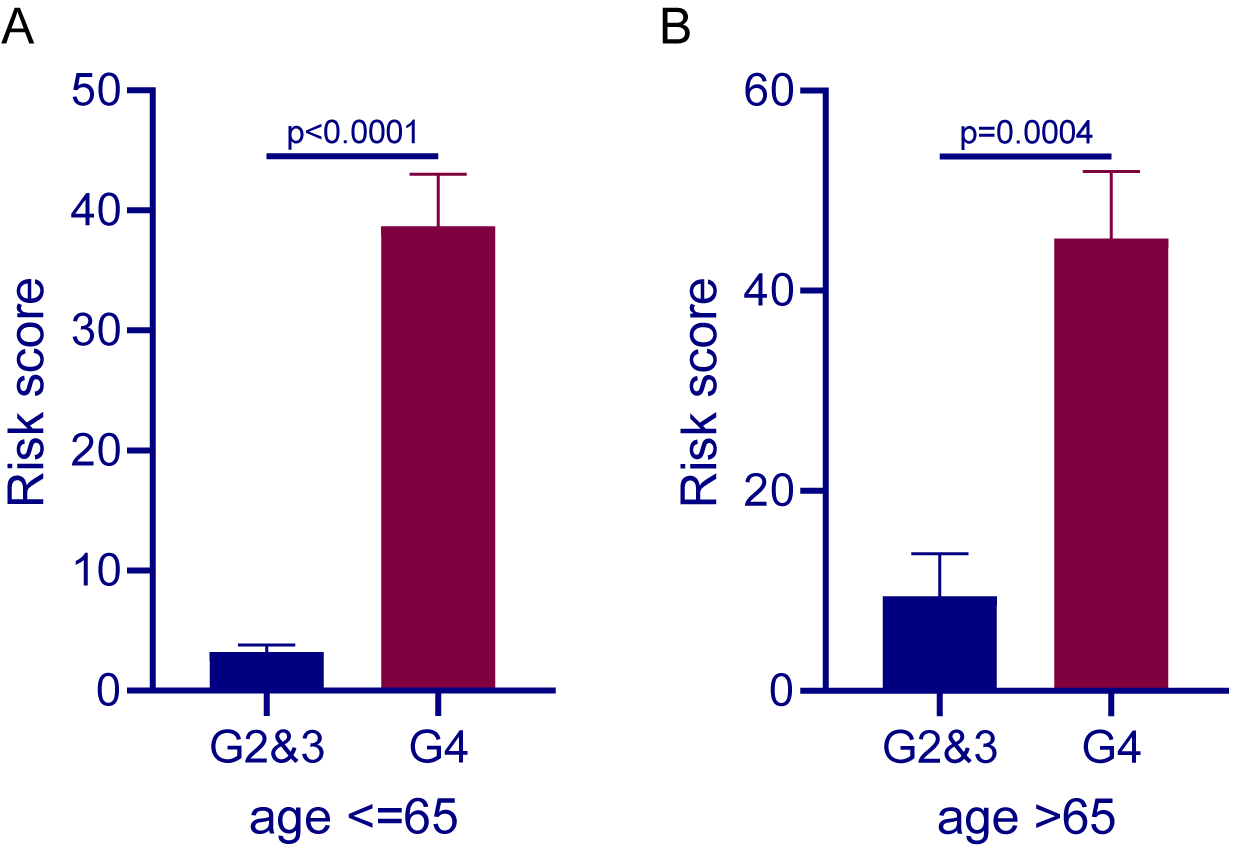

Supplement: Supplementary file 1 [file Image3.tif]

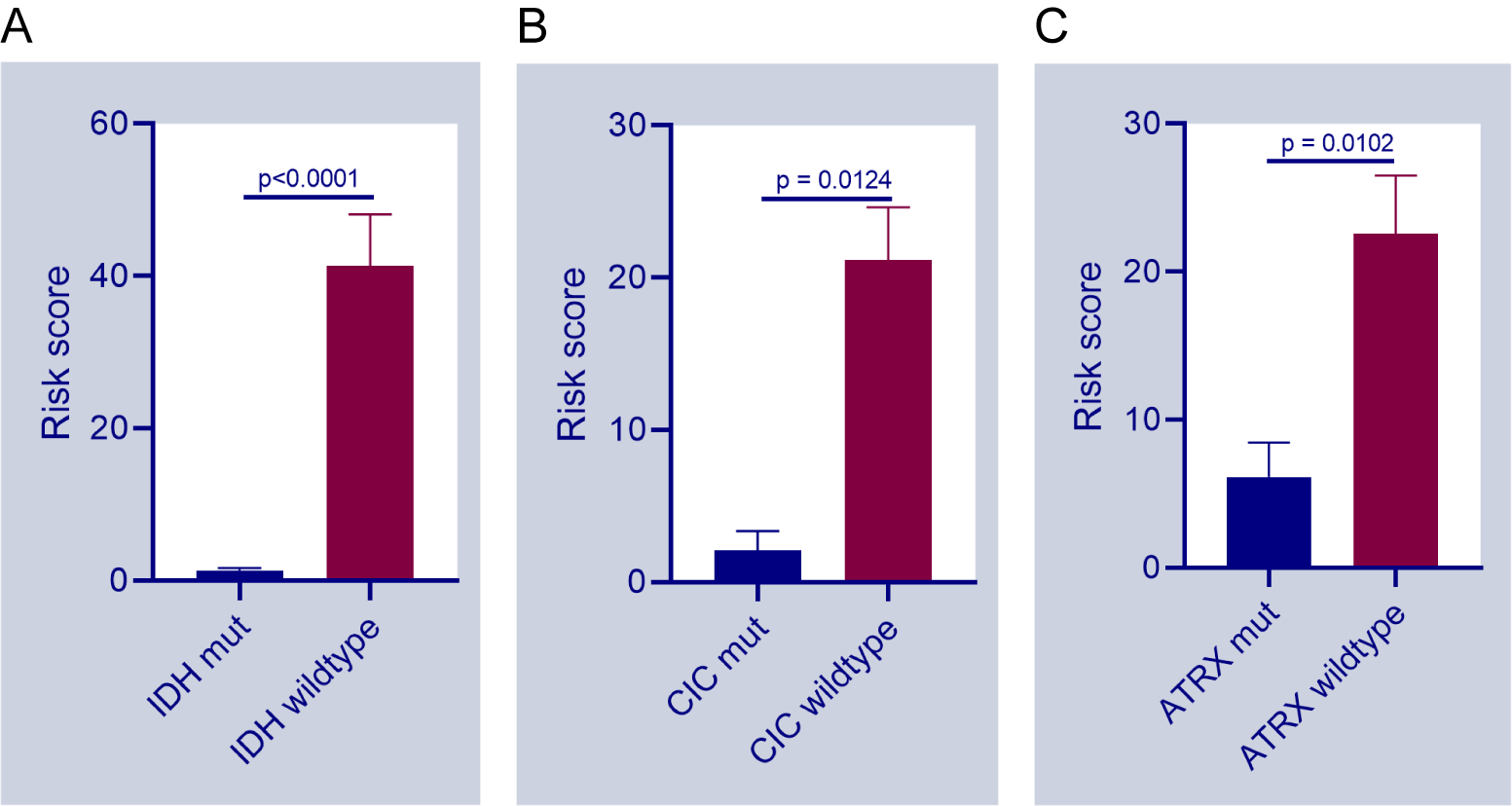

Supplement: Supplementary file 2 [file Image4.tif]

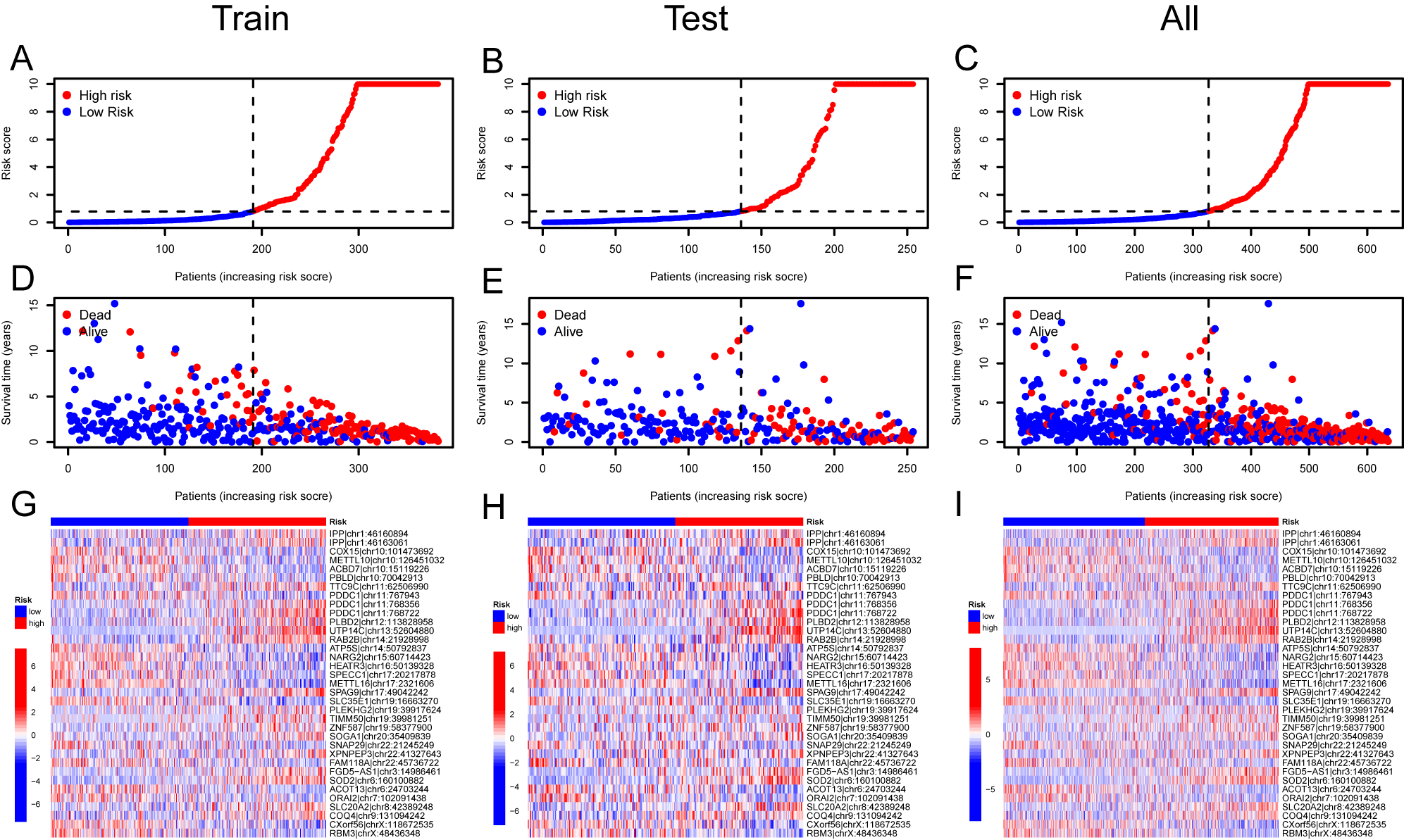

Supplement: Supplementary file 4 [file Image2.tif]

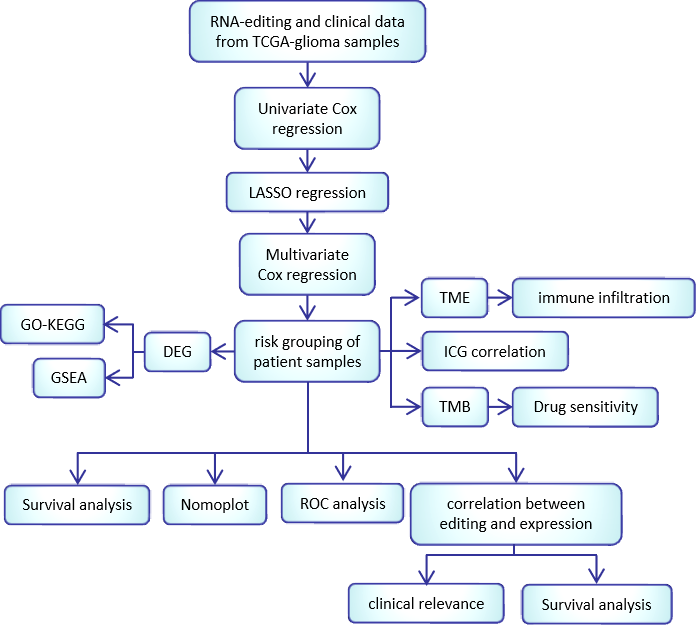

Supplement: Supplementary file 5 [file Image1.tif]
